# Synthesis, Optimization and Molecular Self-Assembly Behavior of Alginate-g-Oleylamine Derivatives Based on Ugi Reaction for Hydrophobic Drug Delivery

**DOI:** 10.3390/ijms25158551

**Published:** 2024-08-05

**Authors:** Yanan Bu, Xiuqiong Chen, Ting Wu, Ruolin Zhang, Huiqiong Yan, Qiang Lin

**Affiliations:** 1 Key Laboratory of Tropical Medicinal Resource Chemistry of Ministry of Education, College of Chemistry and Chemical Engineering, Hainan Normal University, Haikou 571158, China; buynedu@163.com (Y.B.); chenxiuqiongedu@163.com (X.C.); wt09250916@163.com (T.W.); 13021328869@163.com (R.Z.); linqianggroup@163.com (Q.L.); 2 Key Laboratory of Water Pollution Treatment & Resource Reuse of Hainan Province, College of Chemistry and Chemical Engineering, Hainan Normal University, Haikou 571158, China; 3 Key Laboratory of Natural Polymer Functional Material of Haikou City, College of Chemistry and Chemical Engineering, Hainan Normal University, Haikou 571158, China

**Keywords:** algal polysaccharides, polysaccharide derivatives, micellar aggregates, Ugi four-component reaction, hydrophobic pharmaceutical formulations

## Abstract

To achieve the optimal alginate-based oral formulation for delivery of hydrophobic drugs, on the basis of previous research, we further optimized the synthesis process parameters of alginate-g-oleylamine derivatives (Ugi-FOlT) and explored the effects of different degrees of substitution (DSs) on the molecular self-assembly properties of Ugi-FOlT, as well as the in vitro cytotoxicity and drug release behavior of Ugi-FOlT. The resultant Ugi-FOlT exhibited good amphiphilic properties with the critical micelle concentration (CMC) ranging from 0.043 mg/mL to 0.091 mg/mL, which decreased with the increase in the DS of Ugi-FOlT. Furthermore, Ugi-FOlT was able to self-assemble into spherical micellar aggregates in aqueous solution, whose sizes and zeta potentials with various DSs measured by dynamic light scattering (DLS) were in the range of 653 ± 25~710 ± 40 nm and −58.2 ± 1.92~−48.9 ± 2.86 mV, respectively. In addition, RAW 264.7 macrophages were used for MTT assay to evaluate the in vitro cytotoxicity of Ugi-FOlT in the range of 100~500 μg/mL, and the results indicated good cytocompatibility for Ugi-FOlT. Ugi-FOlT micellar aggregates with favorable stability also showed a certain sustained and pH-responsive release behavior for the hydrophobic drug ibuprofen (IBU). Meanwhile, it is feasible to control the drug release rate by regulating the DS of Ugi-FOlT. The influence of different DSs on the properties of Ugi-FOlT is helpful to fully understand the relationship between the micromolecular structure of Ugi-FOlT and its macroscopic properties.

## 1. Introduction

In recent years, amphiphilic polymers have been verified to be an extremely important class of polymeric materials that can self-assemble into a variety of functional micro/nanostructures (such as micelles and vesicles) with potential applications in biomedical, biosensor and food applications [1,2]. A variety of natural, semisynthetic and synthetic hydrophilic polymers can be applied to synthesize amphiphilic polymers with different characteristics [3,4]. Among these polymers, natural polysaccharides, mainly derived from animals, plants and microorganisms, are considered to be one of the most prominent and promising candidates for the construction of amphiphilic polymers due to their important properties such as abundant sources, low cost, non-toxicity, good biocompatibility, chemical modifiability and in vivo degradation into harmless products via the enzymatic or non-enzymatic modes [5,6,7,8].

Sodium alginate (SA) is a linear polyanionic polysaccharide composed of two structural units, β-D-mannuronic acid (M) and its C-5 isomer α-L-guluronic acid (G), which are irregularly linked by β-(1, 4) glycosidic bonds. Its structure contains three different chain segment blocks, namely, homopolymer M chain segment block (-M-M-M-), homopolymer G chain segment block (-G-G-G-) and heteropoly chain segment block (-G-M-G-M-) [9,10,11]. SA is recognized as a safe natural polysaccharide with significant advantages such as biocompatibility, biodegradability, non-toxicity, non-allergy and easy modification [12,13,14]. Therefore, the development of SA-based amphiphilic polymers for the construction of self-assembled nanocarriers has broad application prospects in the field of drug delivery.

Recently, the Ugi four-component condensation reaction (Ugi-4CR) has attracted the attention of many researchers in polymer chemistry and involves the coupling of four chemical functional groups (aldehyde/ketone, amine, carboxylic acid and isocyanide compounds) [15,16]. In the current literature, most alginate-based polymers are prepared by using flammable liquid cyclohexyl isonitrile with high toxicity, heavy odor and high price, which is not conducive to environmental protection and health, and is not conducive to industrial production [17,18,19]. Through screening the isocyanide compounds currently on the market, it was found that p-phenylmethylsulfonyl isonitrile (p-TI) is a solid that is stable, almost odorless and can be stored at room temperature without decomposition, and its price is the most advantageous among isocyanates [20]. In previous work, our group also tried to substitute p-TI for the toxic cyclohexyl isonitrile to prepare amphiphilic alginate derivatives with good biocompatibility, which were used to construct controlled, poorly soluble drug release systems [21,22,23]. Therefore, using p-TI instead of cyclohexyl isonitrile to prepare amphiphilic polymers is of great research value for the development of polymeric biomaterials.

In the previous work, we preliminarily explored the synthesis of an amphiphilic sodium alginate-g-oleylamine derivative (Ugi-FOlT) with a DS of 20.4% via the Ugi-4CR reaction using SA as a hydrophilic main chain and bio-based oleylamine (unsaturated fatty amine) as a hydrophobic side chain and p-TI instead of cyclohexyl isonitrile [24]. On the basis of previous work, we focused on the influence of different DSs on the physicochemical properties of Ugi-FOlT to further analyze and understand the relationship between the micromolecular structure of Ugi-FOlT and its macroscopic properties, so as to achieve the optimal alginate-based hydrophobic drug formulation. In the present work, we further optimized the preparation process parameters of Ugi-FOlT to achieve its simple and efficient synthesis. Furthermore, the effects of different degrees of substitution (DSs) on the amphiphilic properties, molecular self-assembly properties and self-assembled nanomicelles’ colloidal properties of Ugi-FOlT, and the in vitro cytotoxicity and drug release behavior of Ugi-FOlT were also mainly explored. The objective of this study was to lay a theoretical foundation for the development of optimal alginate-based oral formulations for biomedical applications based on the Ugi-4CR reaction.

## 2. Results and Discussion

### 2.1. Optimization of Process Parameters for the Synthesis of Ugi-FOlT

**Figure 1:**
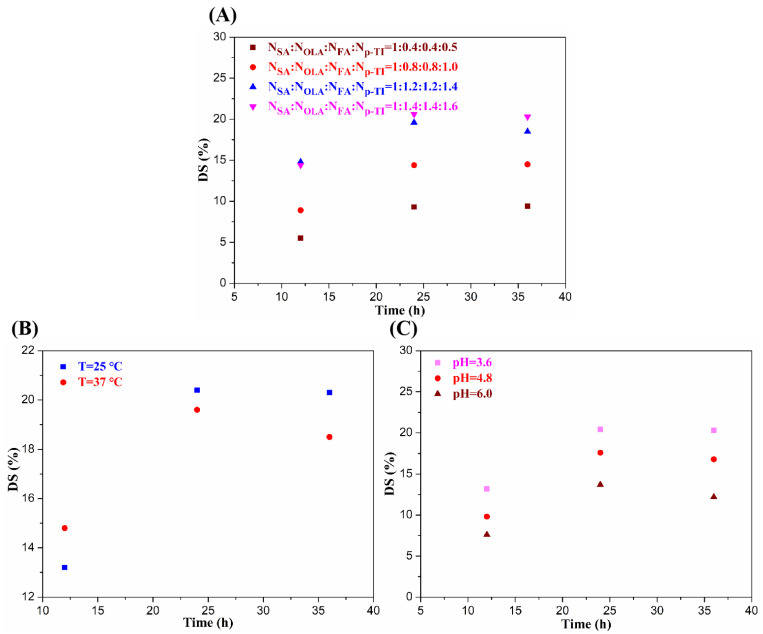
DS as a function of reaction time after coupling of oleylamine to SA varying (**A**) molar ratios of oleylamine to SA at 25 °C with pH value of 3.6, (**B**) temperatures using the feed molar ratio of 1:1.2:1.2:1.4 (N_SA_:N_OLA_:N_FA_:N_p-TI_) with pH value of 3.6 and (**C**) pH values using the feed molar ratio of 1:1.2:1.2:1.4 (N_SA_:N_OLA_:N_FA_:N_p-TI_) at 25 °C.

The Ugi-FOlT was synthesized by UGI-4CR reaction. According to the reaction mechanism of UGI-4CR and considering that p-TI is unstable and decomposes easily in acidic solution, all optimization experiments were based on the same molar amount of formaldehyde and oleylamine and a 20% excess of the p-TI dosage relative to the molar amount of oleylamine to ensure that the reaction could proceed completely [20]. On this basis, the effects of reaction parameters such as molar ratio of raw materials, reaction time, reaction temperature and pH value of solution on the DS of Ugi-FOlT were investigated. Through a series of experiments, the optimum reaction conditions for the preparation of Ugi-FOlT with high DS were determined, with the results shown in Table 1. The effects of reaction parameters such as molar ratio of raw materials, reaction temperature and pH value of solution on the DS of Ugi-FOlT under different reaction times are shown in Figure 1. It can be observed that with the increase in reaction time, the DS of Ugi-FOlT gradually increased and reached the peak value when the reaction was carried out for 24 h. This may be attributed to the fact that SA is a kind of polymer that has a large number of intramolecular and intermolecular hydrogen bonds and has a rigid structure, and the reaction takes a certain amount of time to destroy this structure. When the reaction time was extended to 36 h, the DS of Ugi-FOlT not only did not increase, but presented a slightly decreasing trend, which may mean that too long reaction time leads to a certain degree of degradation of the generated product in the solution, resulting in a slight decrease in its DS. From the point of view of energy saving and efficiency improvement, 24 h was the best reaction time. In addition, the effect of molar ratio of raw materials on the DS of Ugi-FO1T is shown in Figure 1A. When the molar ratio of raw material (N_SA_:N_OLA_:N_FA_:N_p-TI_) gradually increased from 1:0.4:0.4:0.5 to 1:1.2:1.2:1.4, the DS of Ugi-FOlT gradually rose from 9.5% to 20.4% after 24 h reaction. When the molar ratio of the raw material was sequentially increased to 1:1.4:1.4:1.6, the DS of UI-FOLT was 20.5%, and no significant improvement was observed. This phenomenon indicated that the increase in oleylamine and formaldehyde relative to the molar amount of SA was conducive to the enhancement in DS for Ugi-FOlT, but when the dosage of oleylamine and formaldehyde reached a certain amount, there was steric hindrance between the hydrophobic groups on the molecular chain of Ugi-FOlT, which probably affected the further grafting of oleylamine in the reaction. Considering the problem of removing unreacted raw materials in the later stage, we determined that the molar ratio of raw materials for preparing Ugi-FOlT with the highest DS was 1:1.2:1.2:1.4.

Additionally, the reaction temperature affected the DS of Ugi-FOlT; the results are presented in Figure 1B. Within 12 h of the initial reaction, the DS of Ugi-FOlT obtained at 37 °C was 14.8%, which was higher than that at 25 °C (13.2%). When the reaction time was extended to 24 h, the DS of Ugi-FOlT at 25 °C was 20.4%, higher than that at 37 °C (19.1%), which may be attributed to the fact that the high reaction temperature was beneficial to improving the reaction rate, resulting in a higher DS of Ugi-FOlT at the initial stage. However, high reaction temperature would cause unnecessary side reactions, which was not conducive to increasing the DS of the products. Therefore, the optimum temperature for the synthesis of Ugi-FOlT reaction was 25 °C. Finally, the pH value of the solution also had a great influence on the DS of Ugi-FOlT. The effect of solution pH on the DS of Ugi-FOlT is displayed in Figure 1C. When the pH value of the solution was lower than 3.6, the alginate in the solution was protonated to form alginic acid that was insoluble in water, which is detrimental to the reaction [25]. However, when the pH value of the solution was increased, the DS of Ugi-FOlT displayed a decreasing trend; especially when the pH value of the solution was adjusted to 6.0, the DS of Ugi-FOlT decreased significantly. This was owing to the fact that the increase in the pH of the solution is not conducive to the protonation of the reaction intermediate imide. In addition, when the pH value of the solution was high, the carboxyl groups on the SA skeleton mainly existed as carboxyl anions, and the protonated carboxyl groups were less, resulting in their failure to fully participate in the Ugi-4CR reaction. Therefore, the optimal pH for the reaction was 3.6.

In short, the process conditions for the preparation of Ugi-FOlT with high DS were obtained through the experiment, that is, the molar ratio of the raw material (N_SA_:N_OLA_:N_FA_:N_p-TI_) was 1:1:2:1.2:1.4, the pH value of the solution was 3.6, the reaction temperature was 25 °C and the reaction time was 24 h. In order to explore the effects of different DSs on the physicochemical properties and drug release properties of Ugi-FOlT, we selected Ugi-FOlT with DS of 9.5%, 14.6% and 20.4% for follow-up experimental studies, and the corresponding derivatives were, respectively, named as Ugi-FOlT9.5, Ugi-FOlT14.6 and Ugi-FOlT20.4.

### 2.2. Structural Characteristics of Ugi-FOlT

As a hydrophobic segment, the oleylamine was grafted on the hydrophilic SA skeleton by UGI-4CR reaction. The contents of C, H and N elements in Ugi-FOlT were determined by the elemental analyzer. The contents of various elements in Ugi-FOlT9.5, Ugi-FOlT14.6 and Ugi-FOlT20.4 are shown in Table 2. As seen, the content of N element in raw material SA was close to zero, while the content of N element in Ugi-FOlT grafted with oleylamine significantly increased, so it can be speculated that the oleylamine reacted with SA in Ugi-FOlT via the Ugi-4CR reaction.

**Figure 2:**
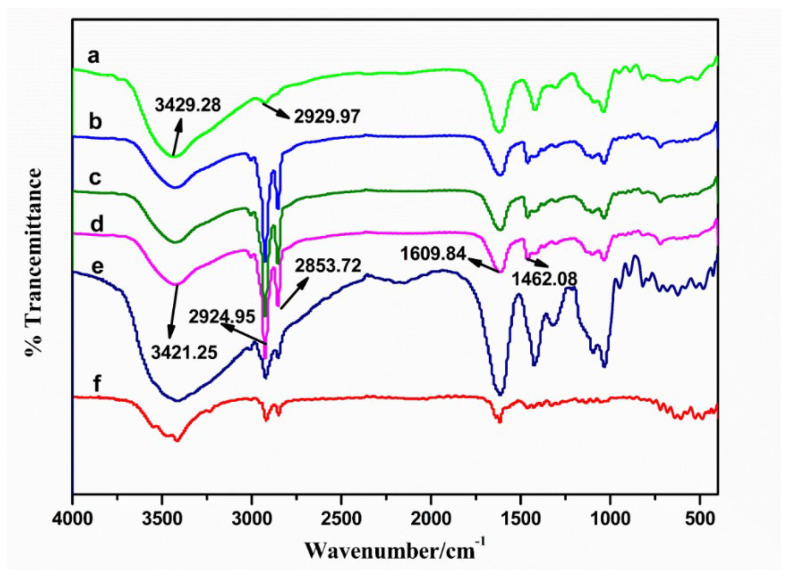
FT-IR spectra of (**a**) SA, (**b**) Ugi-FOlT9.5, (**c**) Ugi-FOlT14.6, (**d**) Ugi-FOlT20.4, (**e**) OLA and (**f**) SA + OLA mixture.

Figure 2 shows the typical FT-IR of SA, OLA, SA + OLA mixture, and Ugi-FOlT. The raw SA exhibited a strong and wide absorption peak at 3429.28 cm^−1^, which was attributed to the stretching vibration of hydroxyl groups. The two peaks located at 1620.94 and 1419.62 cm^−1^ belonged to the asymmetric and symmetrical stretching vibrations of carboxyl groups, respectively. Meanwhile, the peaks appearing at 2929.97 and 1034.54 cm^−1^ were assigned to the stretching vibrations of C-H and C-O-C in the uronic acid skeleton, respectively [26]. In addition to the characteristic absorption peak similar to that of SA, the large and wide peak for the synthesized Ugi-FOlT ascribed to the hydroxyl stretching vibration at 3421.25 cm^−1^ was sharped, indicating that intramolecular hydrogen bond on the glucuronic acid chain had been destroyed. At the same time, more obvious and extremely sharp absorption peaks appeared at 2924.95 and 2853.72 cm^−1^ owing to the antisymmetric and symmetric stretching vibration of methylenes in the oleylamine molecular structure (CH_3_(CH_2_)_7_CH=CH(CH_2_)_8_NH-) [27]. The above results showed that oleylamine, formaldehyde and p-TI were successfully grafted onto SA’s molecular skeleton, thus generating Ugi-FOlT by Ugi-4CR reaction.

**Figure 3:**
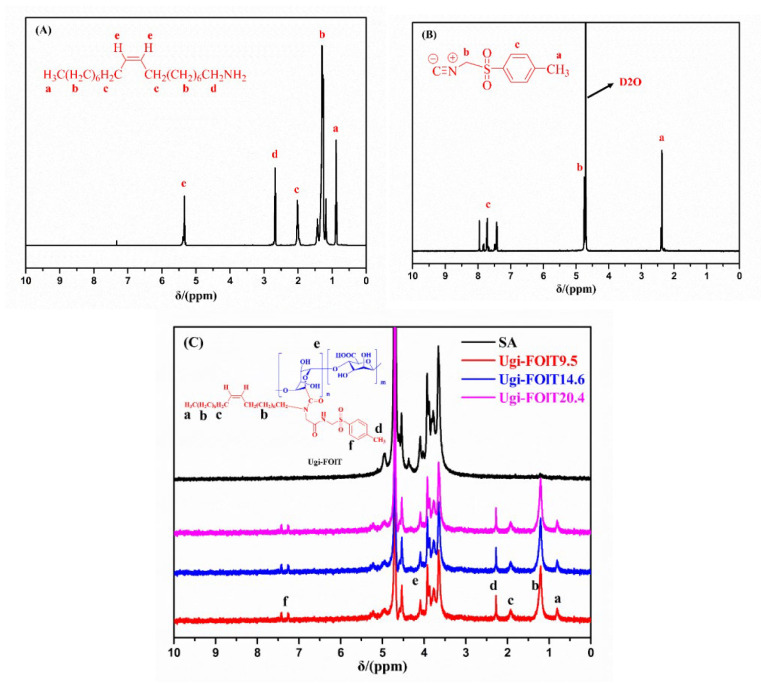
^1^H NMR spectra of (**A**) OLA (CDCl_3_), (**B**) TOSMIC (D_2_O) and (**C**) SA, Ugi-FOlT9.5, Ugi-FOlT14.6 and Ugi-FOlT20.4 (D_2_O).

The chemical structure of Ugi-FOlT can be further confirmed by ^1^H NMR (400 MHz) analysis. ^1^H NMR spectra of oleylamine (CDCl_3_), p-TI (D_2_O), SA (D_2_O) and Ugi-FOlT (D_2_O) are shown in Figure 3. The strong proton signal peak at δ 4.7 ppm belongs to the proton peak of solvent D_2_O. The proton signal peak at δ 3.6~5.1 ppm is attributed to the proton peak on the SA skeleton [28,29]. By comparison, it was found that the signal peaks of protons on the molecular skeleton of SA were retained in the spectra of Ugi-FOlT, indicating that the main skeleton structure of SA was retained in its structure during the modification. In addition, Ugi-FOlT revealed some additional proton signal peaks in the δ 7.0~8.0 ppm and δ 0.6~3.0 ppm ranges. Among them, the proton signal peak at δ 7.41 and 7.27 ppm was due to the proton peak on the benzene ring of p-TI, and the proton signal peak at δ 5.22 ppm was ascribed to the proton peak on the oleylamine’s double bond (-CH=CH-). The proton signal peak at δ 2.27 ppm was attributed to the proton peak on methyl (-CH_3_) of p-TI, and the proton signal peak at δ 1.92 ppm was assigned to the proton peak on the double-bonded methylene (=C-CH_2_-) of oleylamine. The proton signal peaks at δ 1.3~1.2 ppm and 0.81 ppm were owing to the proton peaks of oleylamine’s methylene (-CH_2_-) and methyl (-CH_3_), respectively [30]. The results further confirmed that formaldehyde, oleylamine and p-TI successfully reacted with SA via the Ugi-4CR reaction.

**Figure 4:**
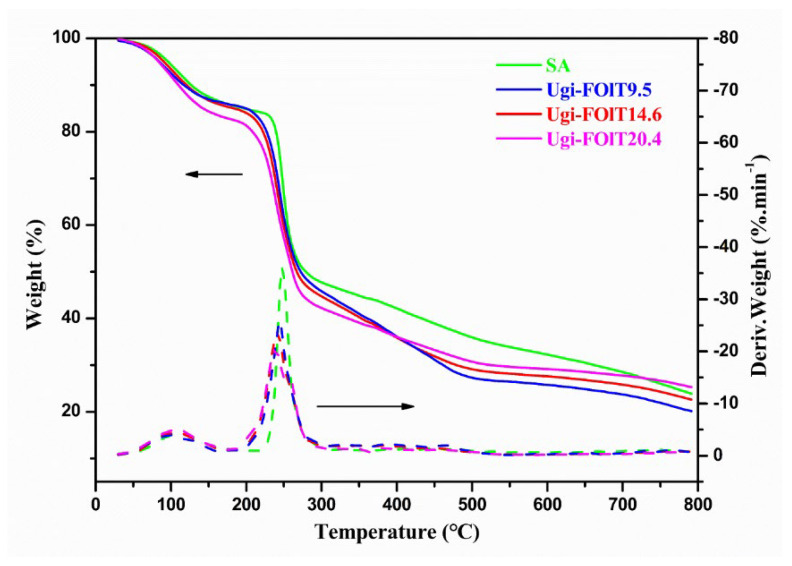
Thermogravimetric analysis and derivative thermograms of SA, Ugi-FOlT9.5, Ugi-FOlT14.6 and Ugi-FOlT20.4.

TGA is a common thermal analysis method that can be used to determine characteristics such as decomposition reaction, absorbed water content and thermal stability. The thermal stability performance of SA and Ugi-FOlT was analyzed by TGA and DTG measurements. The TGA and DTG curves of SA and UGI-FOLT are shown in Figure 4. It can be seen from the TGA curves that SA and Ugi-FOlT underwent two major stages of weightlessness. Among them, the first weight loss between 50 °C and 150 °C was due to the hydrogen bond between the polar groups (hydroxyl and carboxylic groups) and water, which caused the evaporation of the physical adsorption and wrapped water in the polymer. The second weight loss stage occurred in the range of 200~280 °C, which mainly resulted from the thermal cracking of the polymer molecular chain, which gradually cracked into CO_2_, CO and H_2_O [31,32,33]. When the heating temperature exceeded 500 °C, the polymer lost weight completely and a residue of about 20 wt. % was obtained, which may have been caused by carbides formed after the polymer was calcined at high temperatures.

Moreover, there are two peaks in the DTG curves of SA and Ugi-FOlT, which also indicate that the polymer underwent two major weight loss stages, consistent with the results shown in the TGA curves. In addition, the DTG curves also visually display the initial cracking temperature of the polymer. It can be seen that the initial pyrolysis temperatures of Ugi-FOlT9.5, Ugi-FOlT14.6 and Ugi-FOlT20.4 in the second stage were 240, 238 and 232 °C, respectively, which were lower than that of SA (248 °C), indicating that the introduction of hydrophobic oleylamine group destroyed the intramolecular and intermolecular hydrogen bonds of SA, enhanced the flexibility of the molecular chain and reduced the rigid structure of the polymer, thus decreasing the thermal stability of Ugi-FOlT [34].

**Figure 5:**
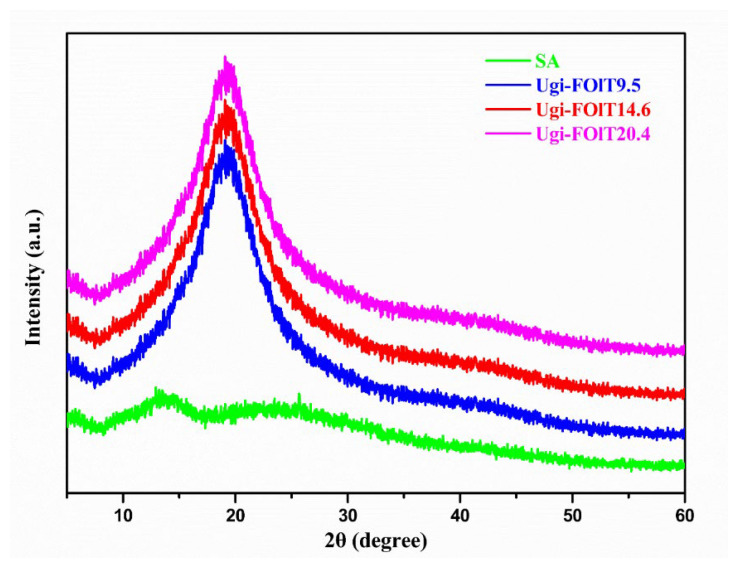
X-ray diffractograms of SA, Ugi-FOlT9.5, Ugi-FOlT14.6 and Ugi-FOlT20.4.

Figure 5 shows the XRD patterns of SA and Ugi-FOlT. The XRD pattern of SA exhibited weak and wide diffraction peaks at 2θ = 14.6° and 21.6°, which are attributed to the diffraction peaks of the hydrated crystal structure of SA [35]. In the XRD pattern of Ugi-FOlT, the diffraction peaks corresponding to SA disappeared, and only a strong diffraction peak in the amorphous region located at 2θ = 20° remained, implying that the Ugi-FOlT was the amorphous structure [10]. This further indicated that SA weakened the intermolecular and intramolecular hydrogen bonds during the grafting of the hydrophobic side chain of oleylamine, reduced the rigidity of the molecular chain and enhanced its flexibility, thus reducing the regularity of the polymer molecular chain and increasing the amorphous region in its structure. However, the DS of Ugi-FOlT had no significant effect on its XRD patterns.

### 2.3. Self-Assembly Behavior of Ugi-FOlT

#### 2.3.1. Amphiphilic Ugi-FOlT’s CMC Value

**Figure 6:**
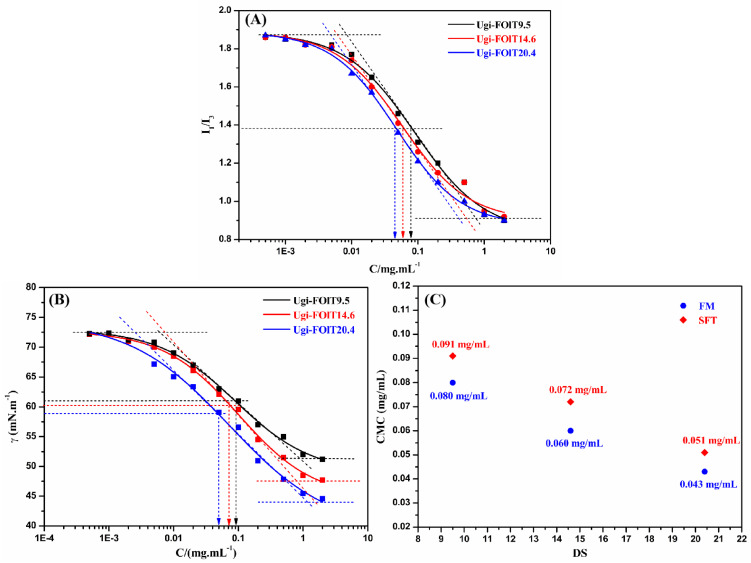
(**A**) Plots of pyrene fluorescence intensity ratio I_1_/I_3_ and (**B**) surface tension as a function of the concentration of Ugi-FOlT at 25 °C; (**C**) CMC values of Ugi-FOlT with different DSs determined by FM and SFT.

Amphiphilic Ugi-FOlT polymers are expected to self-assemble into core–shell micellar aggregates in aqueous solution. In the process of self-assembly, the CMC value is an important parameter for characterizing the self-assembly behavior of amphiphilic polymers, which can be used to characterize the formation and thermodynamic stability of micellar aggregates [36]. The pyrene fluorescence probe method is the most commonly used method to measure the CMC value of amphiphilic polymers. With the increase in polymer concentration, pyrene, as a hydrophobic fluorescence probe, enters the hydrophobic core of the micellar structure through non-covalent interaction forces, and the hydrophilic environment in which it is in solution is transformed into a hydrophobic environment, thus causing sensitive changes in the fluorescence intensity ratio I_1_/I_3_ of pyrene [37]. In other words, the value of I_1_/I_3_ remains basically unchanged when the polymer concentration is lower than the CMC value, but when the polymer concentration reaches the CMC value, the value of I_1_/I_3_ decreases sharply, and the concentration corresponding to the inflection point of the resulting curve is the CMC value of the polymer [38]. The change curves of the ratio I_1_/I_3_ of pyrene fluorescence intensity with the concentration of Ugi-FOlT were shown in Figure 6A, which was similar to our previous results [24]. The CMC values of Ugi-FOlT9.5, Ugi-FOlT14.6 and Ugi-FOlT20.4 obtained by nonlinear fitting were 0.08, 0.06 and 0.043 mg/mL, respectively. They were much lower than the CMC value (2.30 mg/mL) of sodium dodecyl sulfate (SDS), a commonly used low molecular surfactant. For Ugi-FOlT with different DSs, the CMC value decreased with the increase in the DS, as shown in Figure 6C. This may be attributed to the fact that the increase in the grafting rate of hydrophobic groups in Ugi-FOlT was conducive to enhancing the hydrophobic interactions within and between molecules, and Ugi-FOlT was more likely to self-assemble to form micellar aggregates under strong hydrophobic forces, thus exhibiting lower CMC values [39].

In addition, the surface tension method (SFT) is another method for measuring the CMC value of amphiphilic polymers. When amphiphilic polymers begin to self-assemble to form micellar aggregates in aqueous solution, the surface tension at the water/air interface decreases sharply with the increase of their concentration [40]. Figure 6B displays the variations in surface tension of Ugi-FOlT with their concentration. The inflection point of the curves corresponded to the concentration of the polymer’s CMC. The CMC values of Ugi-FOlT9.5, Ugi-FOlT14.6 and Ugi-FOlT20.4 were 0.091, 0.072 and 0.051 mg/mL, respectively, and the corresponding surface tensions were, respectively, 61.0, 60.5 and 58.5 mN/m. These results are similar to the CMC values determined by the pyrene fluorescence probe method.

#### 2.3.2. Colloidal Properties of Ugi-FOlT Self-Assembled Micelles

**Figure 7:**
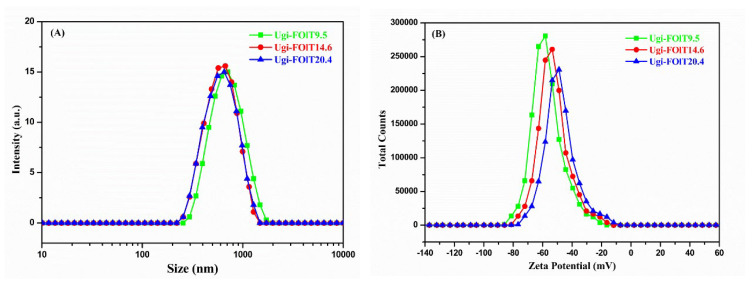
Size (**A**) and zeta potential (**B**) distribution of Ugi-FOlT9.5, Ugi-FOlT14.6 and Ugi-FOlT20.4 micellar aggregates.

The structure of Ugi-FOlT was composed of a hydrophilic SA main chain and hydrophobic oleylamine chain segment. The hydrophobic long carbon chain aggregated inward under the intramolecular and intermolecular hydrophobic forces, and the hydrophilic SA main chain moved outward towards the aqueous solution under the action of hydrogen bonding, etc., and self-assembled to form core–shell micelle aggregates. The particle size, PDI and zeta potential of Ugi-FOlT micelle aggregates were determined by Malvern laser particle size meter, with the results shown in Table 3 and Figure 7.

As shown in Figure 7A, the particle size distribution of Ugi-FOlT micellar aggregates presented relatively wide particle size distribution, ranging from 250 nm to 1300 nm, which was narrower than that of octyl-grafted amphiphilic alginate amide derivative (OAAD) microcapsules (in the range of 100~20,000 nm) [26] and close to that of hexyl alginate ester derivative (HAED) self-aggregates (in the range of 130~1100 nm) [41]. The average particle sizes of Ugi-FOlT9.5, Ugi-FOlT14.6 and Ugi-FOlT20.4 micellar aggregates were 710 ± 40, 670 ± 35 and 653 ± 25 nm (PDI= 0.3~0.4), respectively, which were slightly higher than that of hydrophobically modified alginate derivative (HMAD) self-aggregated micelles [21] and oleoyl alginate ester (OAE) nanoparticles [42]. The main reason for the relatively wide particle size distribution and large average particle size of the above Ugi-FOlT micellar aggregates was that Ugi-FOlT was an ill-defined polymer with the broad molecular weight distribution that had a great influence on the particle size and distribution of its assembled micelles. Meanwhile, some possible micelle aggregation would also increase the hydrodynamic particle size of Ugi-FOlT micellar aggregates after filtration. Furthermore, the particle size of Ugi-FOlT micellar aggregates measured by Malvern laser particle size analyzer is the hydrodynamic particle size in aqueous media, which is defined as the actual diameter of the Ugi-FOlT micellar aggregates plus the size of the water shell formed by interactions between hydrophilic segments and the continuous water phase [43]. The Ugi-FOlT micellar aggregates in solution were surrounded by water molecules and were in an expanded state, making their particle size enlarged [44]. Moreover, Ugi-FOlT possesses negatively charged carboxyl groups, and the electrostatic repulsion of the negatively charged carboxyl groups make the Ugi-FOlT micellar aggregates expand, resulting in an enhancement in their particle size.

The average particle size of Ugi-FOlT20.4 was lower than that of Ugi-FOlT9.5 and Ugi-FOlT14.6, which may be attributed to the stronger hydrophobic and π–π interaction forces of Ugi-FOlT with high oleylamine substitution in aqueous solution, which led to the formation of more compact micelle aggregates [45,46].

A large number of reports have shown that micellar aggregates with higher zeta potential are less likely to agglomerate due to larger electrostatic repulsion between particles, and when the absolute value of the zeta potential of micellar aggregates in solution is higher than 30 mV, they are considered to have good stability [47]. In addition, micellar aggregates with negatively charged surfaces can avoid binding with proteins and cells in the body, which is conducive to extending their circulation time in the body [48]. On the contrary, the cell internalization of such particles would be impeded since the cell membrane is also negatively charged. The zeta potential distribution on the surface of Ugi-FOlT micellar aggregates is shown in Figure 7B. The zeta potential values of Ugi-FOlT9.5, Ugi-FOlT14.6 and Ugi-FOlT20.4 micellar aggregates were −58.2 ± 1.9, −53.5 ± 2.1 and −48.9 ± 2.8 mV, respectively, revealing high negative potential values. This was due to the presence of a large number of −COO^−^ groups on the SA molecular skeleton. The results implies that the Ugi-FOlT micellar aggregates possessed good stability to some extent.

**Figure 8:**
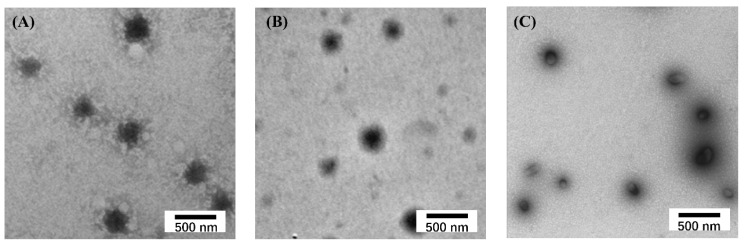
TEM images of (**A**) Ugi-FOlT9.5, (**B**) Ugi-FOlT14.6 and (**C**) Ugi-FOlT20.4 micelle aggregates.

Figure 8 presented the TEM image of Ugi-FOlT micelle aggregates. It was observed that Ugi-FOlT exhibited spheroidal structures with different sizes in aqueous solution, indicating that the hydrophobic modified polymers were amphiphilic and able to self-assemble into micellar structures in aqueous solution. The particle size of Ugi-FOlT9.5, Ugi-FOlT14.6 and Ugi-FOlT20.4 micelle aggregates measured by TEM were 314, 355 and 362 nm, respectively, which were lower than that measured by DLS. This phenomenon was attributed to the fact that the particle size observed by TEM was the actual size of micelle aggregates in the completely dry state, and dry Ugi-FOlT micelle aggregates would shrink due to water evaporation. However, the particle size of Ugi-FOlT micellar aggregates measured by DLS is the hydrodynamic diameter of Ugi-FOlT micelle aggregates in solution, which were surrounded by water molecules, making them in the expanded state; the electrostatic repulsion of the negatively charged carboxyl groups made the Ugi-FOlT micellar aggregates expand, resulting in the enlarged particle size [44].

**Figure 9:**
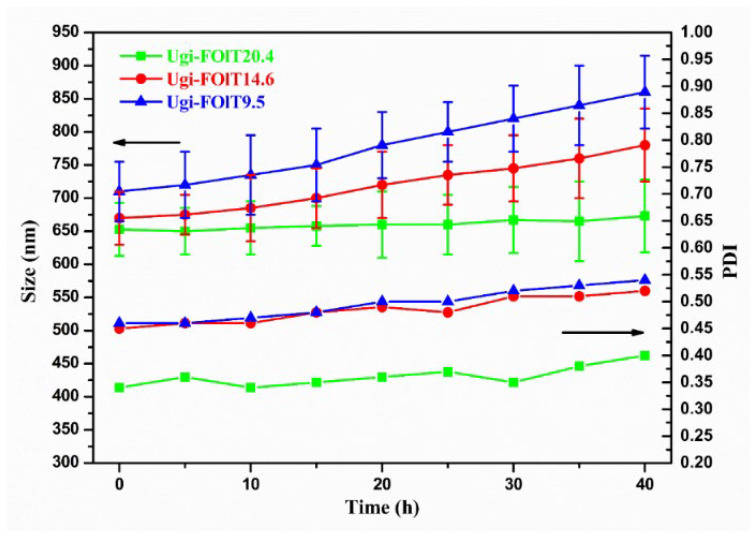
Size distribution of Ugi-FOlT micellar aggregates incubated in PBS at pH 7.4 for 40 h.

**Figure 10:**
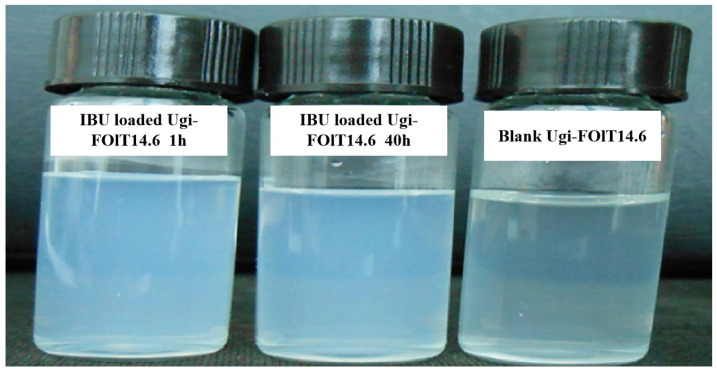
Optical graphs of blank Ugi-FOlT14.6 and IBU loaded Ugi-FOlT14.6 micellar aggregates incubated in PBS at pH 7.4 for 1 h and 40 h.

The stability of Ugi-FOlT micelle aggregates in dispersive media can be analyzed by measuring the change in particle size with time. As shown in Figure 9, after incubating in PBS at pH 7.4 for 40 h, the particle sizes of both Ugi-FOlT9.5 and Ugi-FOlT14.6 micellar aggregates increased to varying degrees, while the particle sizes of Ugi-FOlT20.4 micellar aggregates increased slightly. The results show that the stability of the Ugi-FOlT20.4 micellar aggregates was better than that of Ugi-FOlT9.5 and Ugi-FOlT14.6 micellar aggregates, which may be attributed to the existence of more hydrophobic interactions between the hydrophobic chains of Ugi-FOlT with high DS, which was helpful in the formation of a more compact hydrophobic core, thereby enhancing the stability of its micellar aggregates [23]. Additionally, the optical graphs of blank Ugi-FOlT14.6 and IBU loaded Ugi-FOlT14.6 micellar aggregates incubated in PBS at pH 7.4 for 1 h and 40 h are displayed in Figure 10. It can be found that the drug-loaded Ugi-FOlT14.6 micelle aggregate solution exhibited a darker color than the blank Ugi-FOlT14.6 micelle aggregate solution, and after 40 h incubation, the appearance of the Ugi-FOlT14.6 micelle aggregate solution had no change, revealing its excellent stability.

### 2.4. In Vitro Release Behavior of IBU for Ugi-FOlT Micelles

IBU is an early-developed non-steroidal antipyretic, analgesic and anti-inflammatory drug of phenylpropionic acid, which is the only recommended antipyretic drug for children by the WHO and the US FDA [31]. As a kind of hydrophobic model drug used in the preparation of oral formulations, IBU is easily soluble in organic solvents such as ethanol, ethylene glycol, propylene glycol and ether, but its solubility in water is very low. Poor water solubility may be one of the reasons affecting the absorption of IBU. Amphiphilic Ugi-FOlT could self-assemble into micellar aggregates with hydrophobic core and hydrophilic shell in aqueous solution, and IBU was able to be embedded in its hydrophobic microregion through physical adsorption and interaction between hydrophobic chains to achieve solubilization effect. The encapsulation efficiencies (EE) of Ugi-FOlT9.5, Ugi-FOlT14.6 and Ugi-FOlT20.4 micellar aggregates were 78.24%, 80.65% and 81.30%, respectively. It is known that the EE values of micellar aggregates are directly affected by the concentration of Ugi-FOlT, the mass ratio of IBU/Ugi-FOlT and the DS of Ugi-FOlT [23,44]. Among them, the DS of FOlT involved the grafting of hydrophobic side groups that reflected the affinity with hydrophobic anti-inflammatory drugs, which has the greatest influence on the loading of hydrophobic drugs [41]. With the increase in FOlT’s DS, the EE values of micellar aggregates increased, which indicated that the hydrophobic inner cavity constructed by more hydrophobic side groups was more conducive to the encapsulation of drug.

**Figure 11:**
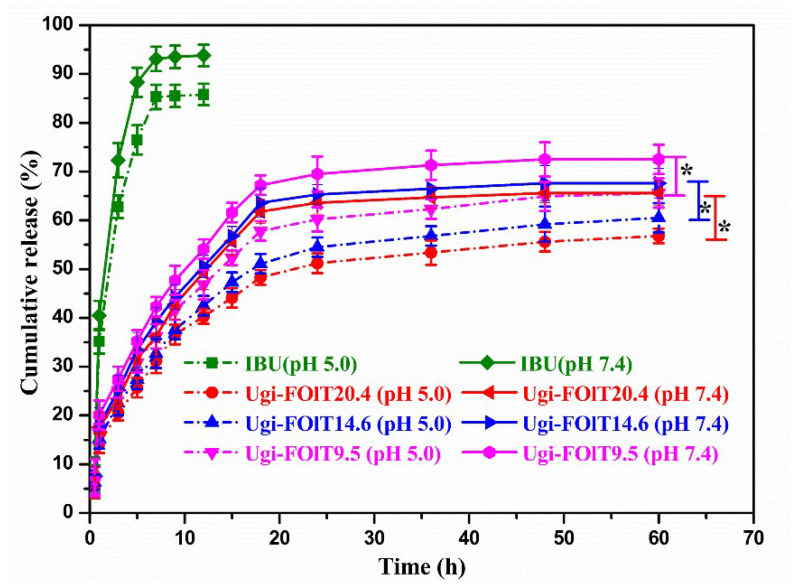
In vitro cumulative release profiles of free IBU and IBU loaded Ugi-FOlT micellar aggregates in PBS (pH 7.4, 5.0) containing Tween 80 (0.5%, *w*/*v*) at 37 °C. * *p* < 0.05: significantly different between pH 7.4 and 5.0 groups at 60 h.

The in vitro drug release behavior of Ugi-FOlT micellar aggregates of IBU under simulated physiological conditions was analyzed by dialysis [49]. The cumulative release curves of IBU for Ugi-FOlT micellar aggregates in PBS at 37 °C with pH 5.0 and 7.4 are shown in Figure 11. The results show that the drug-loaded micelle aggregates could release the drug slowly and continuously in PBS, but the pH value of the releasing medium and the DS of Ugi-FOlT had a certain effect on the cumulative release rate of IBU.

In the PBS release medium with pH 7.4, the cumulative release rate of free IBU reached up to 93% within 7 h, which was attributed to the fact that free IBU was released only by the concentration gradient of the drug, while the concentration of free IBU in the dialysis bag was higher, so the release rate was faster. Within 3 h after the initial release of Ugi-FOlT micellar aggregates, about 23.4%~27.5% of the release amount was achieved by the free diffusion of IBU physically adsorbed on or near the surface of the micellar aggregates, and the release rate of IBU in this part was faster. The rest of the IBU distributed in the hydrophobic core of micelle aggregates needed to break down the hydrophobic force through concentration difference and release slowly and continuously along with the degradation and dissolution of the carrier material, so the cumulative release rate within 7 h was only 36.2%~42.3% [50]. In the sustained release after 3 h, the release rate of IBU in the Ugi-FOlT micellar aggregates was lower than that in the Ugi-FOlT14.6 and Ugi-FOlT9.5 micellar aggregates, which may be due to the self-assembly of Ugi-FOlT20.4 in aqueous solution to form more compact micellar aggregates. Its hydrophobic cavities were more hydrophobic towards IBU molecules, resulting in a slower rate of IBU release [51].

In addition, the cumulative release rate of IBU for Ugi-FOlT micellar aggregates in PBS with pH 7.4 was significantly higher than that in PBS with pH 5.0 at 60 h. This may be attributed to the higher degree of deprotonation of carboxyl functional groups on the Ugi-FOlT molecular chains in the alkaline release medium, which enhanced the hydrophilicity of micellar aggregates and made it easier to absorb water and expand. In addition, due to the formation of more negative carboxylate ions on the molecular chains of Ugi-FOlT, the increase in inter-chain repulsion would cause the polymer chain to form a tensile conformation, resulting in the relaxation of the polymer chains and the destruction of hydrophobic microregions, resulting in an accelerated release rate. However, in the more acidic release medium, more compact micellar aggregates were formed due to the protonation of carboxyl groups on the molecular chains of Ugi-FOlT, which slowed down the release rate of IBU. These results indicate that Ugi-FOlT micellar aggregates possessed a certain pH-responsive release behavior, which could be used as an effective oral formulation. Moreover, it was feasible to control the drug release rate by regulating the DS of hydrophobic segments in amphiphilic Ugi-FOlT.

### 2.5. In Vitro Cytotoxicity of Ugi-FOlT

**Figure 12:**
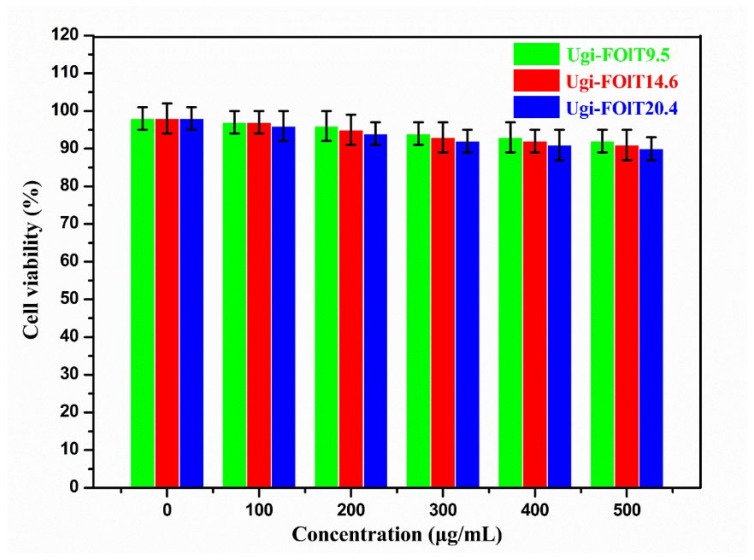
In vitro cytotoxicity of Ugi-FOlT at various concentrations against RAW 264.7 cells after incubation for 48 h.

Due to the special core–shell structures, the Ugi-FOlT micellar aggregates could trap hydrophobic drugs in their hydrophobic cores, which has a wide range of application prospects in drug delivery for oral formulations. However, the polymer micellar aggregates must have good biocompatibility to realize their applications in the biomedical field. The murine macrophage RAW264.7 cells are considered as a representative cell line for various types of macrophages routinely used in many studies. Since RAW264.7 cells have been reported to retain many of the characteristics of macrophages in vivo that have an important role in innate immune defense against microbial infections, which involves secretion of pro-inflammatory mediators and phagocytic activities, we used RAW264.7 cells to evaluate the cytotoxicity of Ugi-FOlT for oral formulations [52]. In this study, the MTT method was used to examine the biological toxicity of Ugi-FOlT with various DSs on RAW 264.7 macrophages, with the results displayed in Figure 12. As seen, after the Ugi-FOlT had been incubated with RAW 264.7 cells for 48 h in the concentration range of 0~500 μg/mL, the cell survival rate decreased slightly with the increase in polymer concentration but remained above 90%. This result indicates that Ugi-FOlT with various DSs had low cytotoxicity to RAW 264.7 cells, exhibiting good biocompatibility [53].

## 3. Materials and Methods

### 3.1. Materials

Sodium alginate (SA, Mw = 135,600 Da, Mn = 102,000 Da) was prepared by NaOH neutralization of alginate based on our previous method and the molecular weight was measured by gel permeation chromatography (GPC, Waters e2695, Santa Barbara, CA, USA) [11]. p-phenylmethylsulfonyl isonitrile (p-TI), oleylamine (90%), formaldehyde (37 wt. % in H_2_O) and ibuprofen (IBU, 98%) were purchased from Shanghai Aladdin Chemical Reagent Co., Ltd. (Aladdin, Shanghai, China). Other reagents were obtained from Guangzhou Chemical Reagent Co., Ltd. (Guangzhou, China). They were of analytical grade and used without further purification. RAW 264.7 cells and biochemical reagents used in the experiments and assays for cytotoxicity were obtained from Gibco (Thermo Fisher Scientific, Carlsbad, CA, USA).

### 3.2. Synthesis and Optimization of Process Conditions of Ugi-FOlT

**Scheme 1:**
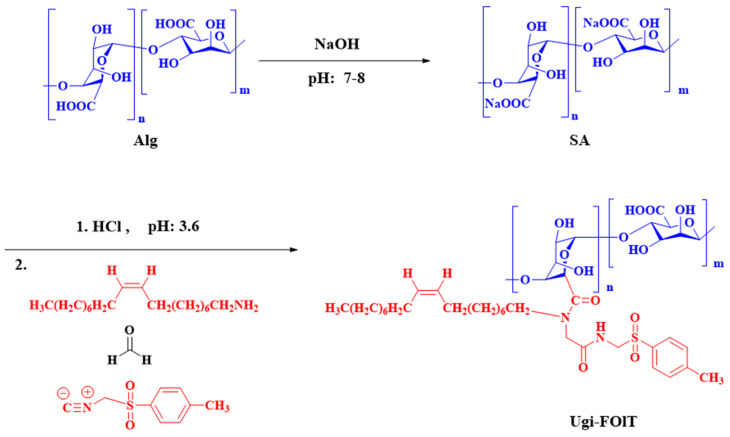
Schematic illustration of synthesis routes of Ugi-FOlT.

The synthesis of sodium alginate-g-oleylamine polymers (Ugi-FOlT) was performed as we previously reported [10]. The Ugi-4CR reaction of formaldehyde, oleylamine, SA and p-TI was carried out using a one-pot method at room temperature, and the schematic synthesis routes are shown in Scheme 1.

In detail, 1.0 g (5.05 mmol) of SA was precisely weighed and completely dissolved in 80 mL deionized water under mechanical agitation in a three-orifice flask to prepare the SA aqueous solution with a mass fraction of 1.25%, and then the pH of this solution was adjusted to 3.6 by dropping a certain amount of 0.1 mol/L HCl aqueous solution. Subsequently, 1.01 mL (6.1 mmol) oleylamine and 0.46 mL (6.1 mmol) formaldehyde solutions were pre-added to a round-bottom flask, which were magnetically stirred at room temperature for 30 min before being added to the prepared aqueous SA solution under mechanical agitation. Then, 1.38 g (7.1 mmol) p-TI was incorporated into the reaction solution. After continuous stirring at room temperature for 24 h, 5 times the volume of absolute ethanol was introduced to the reaction solution to completely precipitate the reaction product. After centrifugation, the precipitate was loaded into a dialysis bag with MWCO 3500 and dialyzed against deionized water for 3 days, and the deionized water was replaced three times a day. The final dialysis-purified product was freeze-dried to obtain dried Ugi-FOlT.

The degree of substitution (DS) of Ugi-FOlT was determined by elemental analysis according to our previous method [24]. The same batch of SA was used to prepare Ugi-FOlT by reacting with oleylamine (OLA), formaldehyde (FA) and p-TI through Ugi-4CR. The effects of reaction parameters such as molar ratio of raw materials, reaction time, reaction temperature and pH value of solution on the DS of Ugi-FOlT were explored via controlling single-factor mode. The DS was used as the target factor to optimize the parameter conditions of the Ugi-4CR reaction in order to achieve simple and efficient synthesis of Ugi-FOlT.

### 3.3. Structural Characterization of Ugi-FOlT

The structure of Ugi-FOlT was characterized by FT-IR, ^1^H NMR, TGA and XRD. FT-IR spectra of the sample was recorded on a Nicolet-6700 (Thermo Fisher Scientific, Carlsbad, CA, USA) with KBr pellets in the range of wavenumbers between 4000 and 400 cm^−1^ for 64 scans with a spectral resolution of 2.0 cm^−1^. ^1^H NMR spectra were recorded at 25 °C using a ULTRASHIELD 400 PLUS spectrometer (Bruker, Berne, Switzerland) operating at 400 MHz with deuterated water (D_2_O) as solvent and tetramethylsilane (TMS) as an internal standard; the concentration of the sample was approximately 8.0~10.0 mg/mL. The thermal stability was studied by means of a 449F3 thermogravimetric analyzer (Netzsch, Selb, Germany) with aluminum crucibles as a sample holder; 8.0~12.0 mg sample was required for the measurement, and the temperature was increased from 25 to 800 °C at a heating rate of 20 °C/min under the N_2_ atmosphere. The XRD pattern of the sample was determined on an AXS/D8 X-ray diffractometer (Bruker, Karlsruhe, Germany) equipped with graphite monochromatized high-intensity Cu-Kα radiation (λ = 0.154 nm, 40 kV, 100 mA), in the range 2θ = 5°~60° and with a scan rate of 5 °/min.

### 3.4. Determination of the Critical Micelle Concentration Value of Ugi-FOlT

According to previous reports, both the pyrene fluorescent probe method and surface tension method can measure the critical micelle concentration (CMC) value of Ugi-FOlT [37].

The fluorescence spectroscopy of Ugi-FOlT was recorded using pyrene as a fluorescence probe on a fluorescence spectrophotometer (F7000 spectrophotometer, Hitachi, Tokyo, Japan); 0.15 mol/L aqueous NaCl solution was used as the solvent to prepare the sample solution, which was beneficial to facilitating the self-assembly of polymer samples. The CMC value can be obtained by plotting the curves of the pyrene fluorescence intensity ratio of the first (373 nm) and the third (384 nm) (I_1_/I_3_) as a function of polymer concentration in the emission spectra of pyrene. This method is regarded as an effective way to determine the self-assembled behavior of amphiphilic polymers. The surface tension measurement of Ugi-FOlT solution with various concentrations was performed on a Dataphysics OCA15EC tension meter (Bruker, Karlsruhe, Germany) using the hanging drop method. The final data were the average value of three measurements.

### 3.5. Preparation and Colloidal Properties of Ugi-FOlT Micelles

The Ugi-FOlT micelles were prepared by directly dispersing Ugi-FOlT in deionized water followed by sonication to facilitate aqueous dispersion and self-assembly. Briefly, the Ugi-FOlT samples were dissolved in distilled water to achieve a micellar solution with the concentration of 0.5 mg/mL at 25 °C. The solution was then sonicated for 4 min by a probe sonicator at 120 W and repeated 3 times to guarantee optically clear dispersion. The sonication pulse was set for 2 s with a waiting time of 4 s between pulses. Finally, the micellar solution was further filtered through a 0.45 μm syringe filter and stored at 4 °C.

The morphology, size and zeta potential of the Ugi-FOlT micelles were examined by TEM and DLS measurement. The morphology of the Ugi-FOlT micelles were examined by a transmission electron microscope (JEM 2100 TEM, JEOL Co., Akishima, Japan) after the sample solution was evaporated on a carbon-coated copper grid. The hydrodynamic diameter and zeta potential of Ugi-FOlT micelles were examined by using a dynamic light scattering meter (Malvern Nano-ZS90 Zetasizer, Malvern, UK) at 25 °C. Additionally, to evaluate the storage stability of the prepared Ugi-FOlT micelles, they were dispersed in PBS (pH 7.4) and shaken at 100 rpm at 37 °C for 5, 10, 15, 20, 25, 30, 35 and 40 h. The stability of the Ugi-FOlT micelles was analyzed by measuring the changes in particle size and PDI with time.

### 3.6. Loading and In Vitro Release of Ibuprofen

The encapsulation of ibuprofen (IBU) into the Ugi-FOlT micelles was achieved by the ultrasound method [54]. A certain amount of IBU was dissolved in anhydrous ethanol to prepare an ethanol solution with a drug concentration of 3.0 mg/mL. Then, 1 mL of this ethanol solution and 2 mL of 2.5 mg/mL Ugi-FOlT micellar solution were mixed well under high speed stirring to form the drug-loaded micelles, followed by dialysis in water to remove the ethanol. In addition, IBU solutions at concentrations of 5.0, 10.0, 15.0, 20.0, 30.0 and 40.0 µg/mL were prepared with PBS at pH 7.4 and 5.0, respectively. The absorbance (Abs) at the maximum absorption wavelength of 222 nm was measured using a U-3900 UV–vis spectrophotometer (Hitachi, Tokyo, Japan). The concentration (C) of IBU was used as the abscissa, and the absorbance value (A) was used as the ordinate for data regression. After linear fitting, its standard curve was obtained, and the results are shown in Table 4. Subsequently, the amount of unencapsulated IBU was measured by the biphasic method [31]. After high-speed centrifugation (12,000 rpm) of the drug-loaded Ugi-FOlT micellar solution, the unencapsulated IBU in the supernatant aqueous solution could be extracted by chloroform. Furthermore, the amount of unencapsulated IBU could be determined by the abovementioned UV–vis spectrophotometer with the standard curve.

So, the Encapsulation Efficiency (EE) for the drug-loaded Ugi-FOlT micelles can be calculated using Equation (1) [31]:

(1) $$Encapsulation\;efficiency\;\left(EE\right)=\frac{Total\;IBU-Unencapsulated\;IBU}{Total\;IBU}\times 100\%$$

The in vitro release behavior of *IBU* from drug-loaded Ugi-FOlT micelles was investigated by a commonly used dialysis method at 37 °C with a medium of PBS buffer (pH 5.0 and 7.4). Tween 80 (0.5%, *w*/*v*) used as a surfactant was added to the PBS media to maintain the sink conditions [55]. Briefly, 1.0 mL of drug-loaded Ugi-FOlT micelles was placed in dialysis bag (MWCO 3500 Da) and then immersed absolutely in 30 mL release medium. The whole release system was incubated in a thermostat water bath and shaken at 100 rpm at 37 °C. At the predetermined time point, 3 mL of the release medium was withdrawn, and the same volume of fresh release medium was then added to maintain the constant volume. The released amounts of *IBU* were measured by UV–vis spectrophotometer with the corresponding standard calibration curves. For comparison, similar release experiments were performed with free *IBU*, with the same amount as found in the drug-loaded Ugi-FOlT micelles. All experiments were performed in triplicate. The percentage of cumulative drug release was calculated from Equation (2):

(2) $$E_{r}=\frac{V_{e}\sum_{1}^{n-1}C_{i}+V_{b}C_{n}}{m_{loaded\;IBU}}\times 100\%$$

where *V_e_* is the volume of sample extracted from the release medium each time (1.0 mL); *V_b_* is the total volume of release medium (30.0 mL); *C_i_* and *C_n_* refer to the concentration of IBU extracted from release medium on the *i*th and nth time, respectively; *n* is the number of times to withdraw the sample solution; *m_loaded IBU_* is the weight of *IBU* previously loaded in the Ugi-FOlT micelles.

### 3.7. Cytotoxicity of Ugi-FOlT

RAW 264.7 macrophages were used for an MTT assay to evaluate the cytotoxicity of Ugi-FOlT in vitro [56]. RAW 264.7 macrophages were first cultured in DMEM supplemented with 10% fetal bovine serum, 100 U/mL penicillin and 100 μg/mL streptomycin in an incubator at 37 °C and 5% CO_2_ saturated humidity. Cells seeded in 96-well plates (4.0 × 10^3^ cells/well) were incubated in an incubator for 24 h. Then, 100 μL of polymer solution (0, 100, 200, 300, 400 and 500 μg/mL) prepared entirely in DMEM was added to the 96-well plate (100 μL DMEM was added in the control group) and incubated for 48 h. Then, 20 μL of MTT solution in PBS (5.0 mg/mL) was added to each well for incubation for 4 h. The medium and MTT solution in the well were removed, and 100 μL of DMSO was added to dissolve the purple methyl chain crystals after washing with PBS. Finally, the 96-well plate was placed on an oscillator for 10 min at low speed, and the absorbance value of each well at a wavelength of 570 nm was measured with a Multiskan MK3 microplate reader (Thermo Fisher Scientific, Carlsbad, CA, USA). The cell viability was calculated by the ratio of the absorbance of the sample to that of the control group with Equation (3):

(3) $$Cell\;viability=\frac{{Abs}_{570\;nm}\;sample-{Abs}_{570\;nm}\;blank}{{Abs}_{570\;nm}\;control-{Abs}_{570\;nm}\;blank}\times 100\%$$

where *Abs*_570 nm_ blank was the absorbance of PBS at 570 nm without cells. *Abs*_570 nm_ sample and *Abs*_570 nm_ control were the absorbance at 570 nm in the presence and in the absence of sample treatment, respectively.

### 3.8. Statistical Analysis

SPSS 12.0 statistical software was applied to process and analyze the data, and the results are expressed as mean ± standard deviation. Single-factor ANOVA was used for comparison of variables, and *p* < 0.05 was considered statistically significant.

## 4. Conclusions

In conclusion, to achieve the optimal hydrophobic oral formulation for biomedical applications, we further optimized the synthesis process parameters of Ugi-FOlT and explored the effects of different DSs on the molecular self-assembly properties and self-assembled nanomicelles’ colloidal properties of Ugi-FOlT, as well as the in vitro cytotoxicity and drug release behavior of Ugi-FOlT. The p-TI was used as the isonitrile component of the reaction to synthesize amphiphilic Ugi-FOlT with SA as the hydrophilic main chain, and oleylamine as the hydrophobic side chain. The synthesized Ugi-FOlT possessed good amphiphilic properties, spherical micellar aggregates with particle sizes in the range of 653 ± 25~710 ± 40 nm were successfully prepared by the ultrasonic-assisted dissolution method, and their zeta potentials ranged from −58.2 ± 1.92 to −48.9 ± 2.86 mV, exhibiting good stability. In addition, the in vitro release of Ugi-FOlT micellar aggregates encapsulated in IBU showed an initial release phase and a continuous slow release process. To note, the carboxyl groups on the molecular skeleton of Ugi-FOlT showed different hydrophilicity at different pH values, which also had a certain effect on the release of IBU in vitro. At the same time, Ugi-FOlT displayed good biocompatibility, with a cell survival rate above 90%. These results indicate that the self-assembled amphiphilic Ugi-FOlT micelles have potential value for the delivery of hydrophobic drugs for oral formulations.

## Figures and Tables

**Table 1:** DS and yield of Ugi-FOlT under different reaction parameters.

N_SA_:N_OLA_:N_FA_:N_p-TI_	Time (h)	Temp (°C)	pH	DS (%)	Yield (%)
1:0.4:0.4:0.5	24	25	3.6	9.5	61.8
1:0.8:0.8:1.0	24	25	3.6	14.6	62.7
1:1.2:1.2:1.4	24	25	3.6	20.4	74.0
1:1.4:1.4:1.6	24	25	3.6	20.5	78.5
1:1.2:1.2:1.4	24	37	3.6	19.1	76.4
1:1.2:1.2:1.4	24	25	4.8	16.4	68.2
1:1.2:1.2:1.4	24	25	6.0	13.1	51.5

**Table 2:** Elemental contents and DS of Ugi-FOlT.

Sample	C (%)	H (%)	N (%)	DS (%)
SA	30.60	5.96	0.005	-
Ugi-FOlT9.5	33.78	7.26	0.87	9.5
Ugi-FOlT14.6	35.45	7.14	1.20	14.6
Ugi-FOlT20.4	38.34	6.97	1.57	20.4

**Table 3:** Sizes, PDI and Zeta potentials of Ugi-FOlT micellar aggregates (*n* = 3).

Sample	Size (nm)	PDI	Zeta Potential (mV)
Ugi-FOlT9.5	710 ± 40	0.38 ± 0.03	−58.2 ± 1.9
Ugi-FOlT14.6	670 ± 35	0.35 ± 0.05	−53.5 ± 2.1
Ugi-FOlT20.4	653 ± 25	0.31 ± 0.04	−48.9 ± 2.8

**Table 4:** Standard curve equations and correlation coefficients of IBU in PBS (pH 7.4 and 5.0).

Solution	λ_max_ (nm)	Standard Curve Equation	R^2^
PBS (pH 7.4)	222	A = 0.04909C + 0.02814	0.99928
PBS (pH 5.0)	222	A = 0.01520C + 0.06280	0.99683

## Data Availability

The original contributions presented in this study are included in the article; further inquiries can be directed to the corresponding author.
